# Supplementary material for: Are carbon emissions trading and green financial instruments synergistic? -Comprehensive quantitative research based on content analysis
Source: PLoS One. 2024 Mar 7;19(3):e0298601. doi: 10.1371/journal.pone.0298601 (PMC10919601; doi:10.1371/journal.pone.0298601)
Supplement: S1 Data — (ZIP) [file pone.0298601.s001.zip › Data/地级市Z_F工作报告.zip!/║╙─╧/░▓╤⌠╩╨/└┤╫╘╣1⁄2╓┌║┼í╛┬φ┐╦╩2╛▌═°í┐_2012.docx]

各位代表：

    现在，我代表市人民政府，向大会报告政府工作，请予审议，并请各位政协委员提出意见。

    一、2011年工作回顾

    2011年是经济形势比较复杂的一年。一年来，在省委、省政府和市委的正确领导下，全市上下深入贯彻落实科学发展观，坚持“四个重在”实践要领，紧紧围绕中原经济区建设，大力实施“八大行动计划”，圆满完成了市十二届人大三次会议确定的各项目标任务，实现了“十二五”良好开局。

    ——综合实力持续攀升。全市生产总值完成1558亿元，增长12.1%，高于全省平均水平0.5个百分点。财政一般预算收入77.4亿元、支出174.5亿元，分别增长18.9%、24.1%。

    ——三大需求持续上扬。全社会固定资产投资929.6亿元，增长28.6%；社会消费品零售总额406.7亿元，增长17.8%；进出口总额18.6亿美元，增长17.8%。

    ——工业经济持续提速。全市规模以上工业增加值805.1亿元，增长18%；实现利润232.4亿元，增长29.2%。4家企业入围全国制造业500强，10家企业入围河南省百强企业。

    ——农业生产持续增收。粮食总产338万吨，连续9年丰产丰收。百万亩优质高效农业发展成效显著。滑县连续9年荣获全国粮食生产先进单位称号。我市被确定为省级农民专业合作社试点市。

    ——人民生活持续改善。城镇居民人均可支配收入18686元，增长14%；农民人均纯收入7586元，增长19.3%，城乡收入均高于全省平均水平。

    一年来，我们突出抓了以下工作：

    （一）坚持把转变发展方式作为首要任务，科学发展模式加快构建。大力实施“传统产业升级行动计划”和“新兴产业倍增行动计划”，成功探索出一条在发展方式转变中统筹兼顾、科学发展的新路子。加快产业集聚区发展。积极推进规划布局调整，加快培育特色产业集群，产业集聚区支撑带动作用不断增强。9个省级产业集聚区完成固定资产投资425.7亿元，实现规模工业主营业务收入1016亿元，5个产业集聚区列入省专业示范产业集聚区重点培育名单。推动产业结构优化升级。大力实施“工业振兴工程”“百亿元园区、百亿元产业、百亿元企业行动计划”，钢铁、装备制造等传统优势产业加快发展，新能源、电子等战略性新兴产业发展势头良好。61个亿元以上项目列入省重点工业结构调整项目。我市被初步列为“十二五”期间全国中西部62个重点老工业基地改造城市之一。提高自主创新能力。连续3次荣获全国科技进步先进市称号，国家知识产权试点市顺利通过验收。成功创建“棉花生物学国家重点实验室”，实现我市国家重点实验室建设零突破。全省首个省级陶瓷砖质量检验检测中心开工建设。加强节能减排。安西循环经济试验区列入省“十二五”规划。环境质量持续改善，化学需氧量、二氧化硫排放量不断削减，万元生产总值能耗下降4.1%。

    （二）坚持把项目带动作为主导战略，发展后劲不断壮大。紧紧抓住投资拉动、扩大内需等关键环节，大力实施项目带动战略，健全完善联审联批联办工作机制，定期组织重点项目督导观摩点评，一批打基础、管长远的重大项目取得突破。129个省、市重点建设项目完成投资209亿元，安钢120万吨冷轧薄板生产线、林州重机高端装备制造业孵化园、宇天化工年产30万吨煤焦油加氢等项目进展顺利，三丰铜业超薄铜板带、中联水泥余热发电、凤宝管业油井管等项目竣工。积极实施“支持服务央属、省属企业行动计划”，健全企业服务长效机制。加大银企对接和融资力度，全年新增贷款和直接融资200亿元。加强煤、电、油、气、运调节和土地管理，有效保障生产要素供给。落实价格监管措施，保持市场物价基本稳定。

    （三）坚持把“三农”工作作为发展命脉，基础地位更加牢固。大力实施“现代农业行动计划”，农业、农村持续良好的发展形势。现代农业蓬勃发展。6大现代农业示范引领区建设全面推进。新增高效农业16万亩，新建大中型规模养殖场113个。新增国家级、省市级重点农业龙头企业46家，新发展壮大农民专业合作社410家。林州市被评为全国生猪调出大县，内黄县荣获全国果蔬标准化建设十强县称号，汤阴县被评为全国农业产业化示范县。农业基础设施全面加强。争取国家、省无偿投资5亿多元，14座水库除险加固工程全部竣工。新建高标准现代化水利方田20万亩。城乡水系一体化建设成效明显，再获省“红旗渠精神杯”。新农村建设稳步推进。新建大中型沼气工程57座，累计解决130万农民生活用气问题。新解决41万农村居民饮水安全问题。农村劳动力培训阳光工程共培训9560人。新改建农村公路215公里。完成53个贫困村整村推进任务，实现5.4万农村人口脱贫。

    （四）坚持把城乡统筹发展作为关键环节，综合支撑功能不断提升。大力实施“加快中心城区建设和城区经济发展行动计划”，积极推进城乡协调发展。城建三年计划成效显著。三年累计实施事关民生和经济发展的项目118个，完成投资400亿元。中华路南北段、西南外环、解放大道道路拓宽改造及地下人防工程等建成投用。安阳迎宾馆、旧城及“城中村”改造等积极推进。洪河、洹河、环城河治理工程顺利实施。安阳新区建设总体方案被省政府正式批复。被评为“国家可再生能源建筑应用示范城市”。我市以甲骨文为元素的安阳展园，荣获第八届中国（重庆）国际园博会金奖。综合交通枢纽初步构建。鹤辉高速公路开工建设，西北环城高速公路、石林高速公路、林长高速公路积极推进。晋豫鲁铁路通道安阳段进展顺利。石武高铁安阳段基本建成。豫东北机场列入国家“十二五”民航发展规划。与新郑机场合作建设安阳空港新城及机场建设框架协议签订。日照港驻安阳办事处及陆路港综合服务中心正式挂牌，陆路港高新区港区建设积极推进。新型城镇化建设扎实推进。深入开展“优胜杯”竞赛活动。113个新型农村社区建设全面启动。15个乡镇入围河南省经济社会发展百强乡镇，水冶镇连续7年保持综合实力第一名。

    （五）坚持把文化旅游作为重要抓手，带动效应逐步显现。坚持“三阳开泰”发展思路，大力实施“服务业提速行动计划”，第三产业快速发展。文化事业全面繁荣。公共图书馆、文化馆、文化站免费开放工作顺利推进。市工人文化宫数字3D影城、巴黎国际影城等数字影院相继开业。圆满完成995个农家书屋建设任务。电视剧《第四片甲骨》、电影《落井的硬币》公映。创作了《曹操立嗣》、《家访》、《森林畅想曲》等优秀文艺作品。旅游业快速发展。成功举办2011中国·安阳殷商文化旅游节、第三届中国（安阳）国际航空运动旅游节等活动。红旗渠、马氏庄园入选全国红色旅游经典景区二期名录。曹操高陵通过国家级重点文物保护单位初评。低空经济和航空运动之都建设迈出坚实步伐。全年接待国内外游客2268.1万人次，增长20.3%；旅游总收入153亿元，增长20.2%。服务业积极推进。放心早餐工程规模进一步扩大，万村千乡市场工程扎实推进，农产品流通体系进一步完善。新建、改建农家店3155家，升级改造农贸市场13个。丹尼斯百货分店、义乌国际商贸城投入运营。欧凯龙、北京王府井百货等知名品牌落户安阳。安阳物流港等现代物流项目进展顺利。

    （六）坚持把改革开放作为根本动力，发展活力不断增强。把改革开放作为持续发展的根本途径，以改革创新破解瓶颈，以开放招商带动全局。各项改革深入推进。37户国企改革任务基本完成。国有和乡镇煤炭企业兼并重组全面实现。成功组建两大钢铁企业集团。林州重机在深圳上市，鑫盛机床、凯瑞数码等6家企业上市工作加快推进。城区管理体制改革积极推进。公共资源交易中心正式运行。集体林权制度主体改革全面完成。财税、医药卫生等改革取得新成效。荣获全国文化体制改革工作先进地区。招商引资领域不断扩大。组织开展了“会战珠三角、集中大招商”，驻地招商，2011华商（安阳）发展投资论坛等一系列大招商活动。与中国建材集团、国药集团、中石化等央企合作进一步深化。河南大通电子无线通讯产业园、美的电器零部件生产基地等一批投资规模大、技术含量高的项目落户安阳。全年引进域外资金390.8亿元，其中，省外资金302.3亿元，实际利用外资2.6亿美元。被评为对外开放工作优秀省辖市。

    （七）坚持把改善民生作为最大责任，群众幸福指数持续提升。筹措资金99.1亿元用于“十项民生工程”和重点支出，75件实事基本完成。就业工作不断加强。新增城镇就业11.7万人，再就业4.3万人，其中就业困难群体再就业1.6万人。农村劳动力转移就业176万人。社会保障日益完善。社会保障标准不断提高，社会保险覆盖面持续扩大，城乡低保实现应保尽保。新建廉租住房5546套，公共租赁住房3697套，经济适用住房3053套，棚户区改造5033套。社会事业协调推进。义务教育取得新成效，城乡薄弱学校办学条件不断改善，基础教育质量明显提升。学前教育三年行动启动实施。安阳师院软件学院开工建设，省卫校、安阳幼师分别升格为河南护理职业学院、安阳幼儿师范高等专科学校，中等职业学校整合工程进展顺利，职教园区粗具规模。成功举办第八届全国特种养殖种植技术成果博览会。创建国家卫生城市圆满成功。市人民医院新院奠基开工，完成2037个标准化村卫生室建设任务。市体育训练中心加快建设。人事人才工作得到加强。低生育水平持续稳定。安全生产形势良好，连续9年被评为安全生产优秀市。食品药品安全保障水平不断提高。积极开展矛盾纠纷排查化解，信访态势保持平稳。深入推进平安安阳建设，社会治安综合防控体系进一步完善。不断深化警务机制改革，大力开展严打整治专项活动，刑事案件发案率大幅下降。加强社会管理和创新，社会大局总体稳定。

    精神文明和民主法治不断加强。以领导方式转变加快发展方式转变，坚持“三具两基一抓手”，深入推进“两转两提”，扎实开展“创先争优”和“提升干部素质、着力为民服务”活动，干部群众思想观念、工作方式、工作作风持续转变，团结奋进、干事创业的氛围更加浓厚。自觉接受人大法律监督、工作监督和政协民主监督，认真办理人大代表议案、建议和政协委员提案。深入推进反腐倡廉建设，不断加强行政效能监察。坚持军民融合发展，积极开展军地互办实事活动，广泛开展全民国防教育和拥军优属、拥政爱民活动，军政军民团结进一步巩固，第五次荣获“全国双拥模范城”称号。不断加强老区建设。依法保护妇女儿童合法权益。社会福利、慈善事业和老龄、残疾人、关心下一代工作健康发展。统计、审计、史志、档案、气象、地震、发展研究、外事侨务、民族宗教、红十字等工作进一步加强。

    各位代表！

    在复杂严峻的形势下取得这些成绩，来之不易。这是市委正确领导的结果，是市人大、市政协大力支持和监督的结果，是全市广大干部群众克难攻坚、共同奋斗的结果。在此，我代表市人民政府，向工作在全市各条战线上的工人、农民、知识分子、干部、解放军指战员、武警官兵、政法干警以及社会各界人士，致以崇高的敬意！向所有关心、支持我市经济社会发展的港澳台同胞、海外侨胞和国际友人，表示衷心的感谢！

    各位代表！

    在肯定成绩的同时，我们也清醒地认识到，经济社会发展仍存在不少矛盾和问题。一是产业结构不优，财税增幅低于全省平均水平，经济发展质量和效益有待进一步提高，转变发展方式的任务仍很艰巨。二是区域发展不协调，城区经济仍然较为滞后。三是受国际国内宏观经济环境和需求不足影响，经济下行压力加大。四是资金难题尚未根本破解，中小企业贷款难没有大的改观，尤其是非法集资现象多发，对我市经济发展和社会稳定造成不利影响。五是一些部门职能转变还不到位，一些干部领导方式、工作方法和运作水平还有待进一步提升。对此，我们一定高度重视，认真解决。

    二、2012年总体要求和奋斗目标

    今年是实施“十二五”规划承上启下的重要一年，也是全面推进中原经济区建设，促进安阳跨越发展的关键一年，做好全年工作意义重大。从总体上看，我们既面临着极为复杂的国内外经济形势挑战，也面临着重大机遇。对我们来说，最大的机遇在于中原经济区上升为国家战略，对于我们争取各方支持更加有利；最大的挑战在于世界经济持续低迷，国内经济下行压力加大，周边城市围绕市场、资金、资源、人才等竞争将更加激烈。因此，我们既要正视困难和挑战，始终保持清醒忧患，更要看到机遇和优势，增强必胜信心，以邓小平理论和“三个代表”重要思想为指导，深入贯彻落实科学发展观，推动经济社会发展取得新的更大的成绩。

    今年政府工作的总体思路是：以科学发展为主题，以转变发展方式为主线，以新型城镇化引领“三化”协调科学发展为路径，以红旗渠精神为动力，紧紧抓住中原经济区建设的重大机遇，坚持“四个重在”实践要领，坚持扭住工业、抓好农业、大力发展第三产业，深入实施项目带动战略，稳中求进，持续提升，改革创新，务实惠民，加快中原经济区区域性中心强市建设步伐。

    今年经济社会发展的主要预期目标是：生产总值增长10%以上，地方公共财政预算收入增长12%，全社会固定资产投资增长21%，社会消费品零售总额增长16%，外贸出口增长18%以上，实际利用外商投资增长20%，城镇居民家庭人均可支配收入增长10%左右，农民人均纯收入增长10%左右，城镇新增就业6.5万人以上，城镇登记失业率4.5%以下，人口自然增长率控制在6.4‰以内，居民消费价格涨幅控制在4%左右，单位生产总值能耗完成省定任务。

    实现上述要求和目标，做好今年各项工作，必须按照中央和省委稳中求进、持续求进的要求，立足安阳实际，围绕“总目标”，把握“总基调”，突出“三化”协调。“总目标”就是保持经济社会发展好的趋势、好的态势、好的气势。“总基调”就是稳中求进、持续提升。“稳”就是保持经济平稳较快发展，保持物价总水平基本稳定，保持社会大局稳定；“进”就是在转变发展方式上取得新进展，在深化改革开放上取得新突破，在改善民生上取得新成效。“持续”就是保持科学发展的意识、思路、举措、进程；“提升”就是调整、优化，在转变中提升，在创新中提升，在开放中提升。突出“三化”协调就是坚持以新型城镇化为引领，以“两个不牺牲”为根本，以城乡统筹为目的的“三化”协调之路。

    《安阳市建设中原经济区区域性中心强市纲要（草案）》、《2012年十项民生工程》已印发各位代表，请一并审议。

    三、2012年工作安排

    今年着重抓好以下十个方面的工作：

    （一）以新型城镇化为引领，提升安阳都市区建设发展水平。新型城镇化引领是“三化”协调发展的关键和突破口，一头连着工业化，一头连着农业现代化。推进“三化”协调发展，必须以新型城镇化为引领，构建城市空间发展新格局。坚持规划先行，加快编制中心城区总体规划、产业规划和土地利用规划，统筹推进各项专项规划、控制性详细规划和重点地区城市设计，实现中心城区规划全覆盖。加快“1＋4＋2”城市组团式发展。“1”就是中心城区，突出主城区和安阳新区的核心带动作用，加快推进以精密铸造、电子信息、物流为主的以产带城的安东新城，以金融、商贸、高科技为主的产城融合的安汤新城，以先进装备制造、电子信息、新能源为主的高新区，以钢铁、化工为主的新西区，以纺织产业为主的洹北新区建设。“4”就是水冶、柏庄、内黄、汤阴四个组团，以高于中心城区的标准，规划建设组团起步区。按照各组团的发展方向、产业定位，着力完善综合交通、现代产业、生态环境、基础设施等支撑体系，实现向中心城区融城发展。“2”就是林州市、滑县两个卫星城，支持其发展成为功能配套的中等城市。积极推进新型农村社区建设。新型农村社区是统筹城乡发展的结合点、推进新型城镇化的切入点、促进农村经济发展的增长点。按照“两分、两力、两业”的原则，坚持“四个率先”、“两个鼓励”、“十个提升”，扎实推进新型农村社区建设。今年年底前，启动25个新型农村社区建设，完成113个新型农村社区建设的年度任务。安阳新区、9个产业集聚区内新型农村社区全部启动。安东新城、安汤新城至少各启动1个3万人到5万人的特大型新型农村社区，各县（市）区至少启动1个1万人以上的大型新型农村社区。

    （二）以新型工业化为主导，加快产业结构优化升级。按照“科技含量高、信息化涵盖广、经济效益好、资源消耗低、环境污染少、人力资源优势得到充分发挥”的标准，坚持做大总量和优化结构并重，持续推进工业振兴工程，为经济发展提供产业支撑。推动产业结构优化升级。继续实施“传统产业升级行动计划”和“新兴产业倍增行动计划”，改造提升传统产业，培育战略性新兴产业，发展新一代信息技术、新能源、生物、高端装备制造、通用航空等产业。重点在钢铁、煤化工、装备制造、食品和纺织等行业谋划实施88个工业升级项目。积极实施光伏产业应用示范工程，规划建设一批光伏发电示范系统、光伏照明示范道路、屋顶光伏电站或光电建筑示范大楼。抓好重大工业项目建设，推进“百亿元园区、百亿元产业、百亿元企业行动计划”初见成效。支持林州市装备制造、安阳县煤化工、滑县电子新能源、内黄县新型陶瓷、汤阴县食品医药、文峰区商贸物流、北关区纺织加工、殷都区钢铁物流、龙安区有色金属等产业做强做大。积极推进产业集聚发展。按照“四集一转”的要求，调整产业集聚区布局，加快产业集聚区整合提升。强化主导产业培育，综合运用规划、土地利用、项目审批、财税调节等手段，推动同类和关联项目集中布局。重点推动集聚区对外连接通道、环保、供电、生活服务等基础设施建设。力争全年完成固定资产投资500亿元，企业营业收入1400亿元。抓好自主创新体系建设。全面实施自主创新体系建设和发展规划，以重点领域优势企业为核心，以市厅会商、知识产权示范市创建为契机，加强产学研合作，强化研发平台和基地建设。持续推进节能减排。严格落实节能减排目标责任制，完善评价考核机制和奖惩制度。大力淘汰落后产能，深入开展企业节能低碳、绿色节能建筑等专项行动。加快安西循环经济试验区建设。继续推进重点区域、重点流域和行业环境综合整治。加大企业帮扶力度。完善重点企业服务制度，积极推动产销对接。对市直工业在政策导向、市场开拓、融资服务、行政审批等方面给予重点帮扶。建立省市重点建设项目、政府投资项目使用市内产品登记制度。加强经济运行调节和监测，保障要素供应。加强物价管理，保持物价总水平基本稳定。

    （三）以新型农业现代化为基础，不断提高农业产业集群发展水平。坚持保粮与增效并重，促进农业生产规模化、专业化、标准化、组织化、集约化、特色化。全面实施高标准粮田“百千万”建设工程。按照“合理规划、综合配套、稳定面积、主攻单产”的原则，规划建设一批百亩方、千亩方和万亩方高标准永久性粮田。促进350万亩粮食生产核心区建设，稳定提高粮食生产能力。大力推进农业产业集群发展。突出基地支持、龙头带动、流通服务、特色高效，选择一批上下连接、就地加工为主的农业龙头企业和大中型专业合作社，在6大现代农业示范区的基础上，打造以瓜菜菌、面品加工、花生油脂、林木加工等为主导的10大农业产业集群。新增高效农业16万亩，新建较大型规模养殖场（区）200个，培育壮大规范化、规模化农民专业合作社300家。新建、扩建大型农业龙头企业10家，配套开发现代化、标准化农产品基地450万亩，创建农业产业化集群示范乡镇和特色农村农业经济先进乡镇40个。切实改善农村生产生活条件。加强农村水电路气等基础设施建设，解决20万人饮水安全问题。新改建农村公路200公里。完成新一轮农村电网改造升级。新增沼气用户1.5万户，新建大中型沼气工程50座，努力创建全国沼气示范市。完成5.05万贫困人口脱贫。加强生态建设。积极创建全国绿化模范城市和国家生态园林城市，加快“266”林业重点工程建设，构筑城市南北“两岭”绿色生态屏障。加快矿区生态恢复治理、煤矿塌陷区和农村土壤修复。

    （四）以招商引资为突破，大力提升对外开放水平。对外开放是决定安阳命运的关键抉择，要坚持以开放促改革、促创新、促发展，推动对外开放不断向更大范围、更宽领域、更高层次迈进。全力推进大招商活动。按照产业结构调整方向和重点，依托产业集聚区，开展园区招商、定向招商和面向央企招商。充分发挥行业商会作用，加强行业对接招商。建立与知名企业合作长效机制，加强与海尔、联邦制药、太极、TCL王牌等企业的联系，谋划实施一批高质量的合作项目。大力开展“招商引资项目落实年”活动，积极推动中建材蚌埠玻璃研究院“三新”产业基地、省投资集团高端电子玻璃产业基地、安化集团煤化工系列项目、凤竹集团纺织产业基地和中国五矿产业园等重大项目落实。积极拓展开放领域。坚持“引进来”与“走出去”相结合，大力推进各领域对外开放。加快服务业对外开放，吸引境内外投资者进入现代物流、金融、旅游、文化等领域。深化农业对外开放，重点引进食品加工、畜牧养殖与精深加工、花卉苗木等基地型项目。扩大基础设施和城乡建设领域对外开放，鼓励各类资本参与城市建设。积极出台有针对性的扶持政策，多渠道、多途径引进一批域外龙头企业和重大项目，加快形成全方位开放格局。不断完善工作机制。健全领导体制，建立联动协作机制，推动开放工作实现更高水平、更大规模、更好效果。强化软实力支撑，谋划和实施专题招才引智活动。加强招商引资绩效评估，优化开放环境，提升服务水平，营造亲商、安商的良好氛围。

    （五）以项目带动战略为核心，进一步夯实经济发展后劲。项目建设是经济社会发展的载体和动力。抓项目就是抓发展，抓住了项目就等于抓住了经济发展的“牛鼻子”。深入实施项目带动战略。突出“早”字，狠抓“快”字，落实“好”字，抓规划、抓运作，抓跟踪、抓推进，抓开工、抓督察，重点抓好200个亿元以上重大项目，100个亿元以上工业升级、服务业项目，100个以上省市重点项目。确保河南红日集团1万吨高性能锂电铜箔、安彩高科1440万平方米电子信息显示超薄基板等33个项目建成投产。加快推进新能光伏50兆瓦非晶硅薄膜太阳能电池、永达1亿只肉鸡屠宰等45个项目建设。开工建设一铸林钢45万吨铸造生产线、红旗渠实业1万辆新能源客车等34个项目。积极推进圣光医药、安阳2×300兆瓦等级燃气蒸气联合循环热电机组等23个项目前期工作。加快推进安阳物流港、北八道物流园区铁路专用线等项目建设。积极谋划推进北京贯辰河南通用航空产业、文化艺术中心等项目建设。着力扩大投资。优化投资结构，提高投资效益，重点安排一批财政主导的基础设施建设和民生项目。积极争取中央、省投资。在扩大市场准入、拓宽融资渠道、加大财税支持、减轻企业负担等方面制定相关政策措施，引导民间投资进入基础设施建设领域。优化项目建设环境。进一步完善重点项目市长办公会议、市级领导分包、联审联批联办等制度，健全项目考核奖惩长效机制。组织开展全市重点项目观摩点评活动。力争新开工项目、竣工项目和年度投资目标100%完成。

    （六）以文化旅游产业为抓手，大力发展现代服务业。发挥文化旅游资源优势，加快建设华夏历史文明传承创新示范市，提升城市文化软实力。繁荣发展文化事业。加强各类文化遗产保护、传承和利用。支持三杨庄汉代遗址申报国家考古遗址公园，继续推进殷墟国家大遗址公园建设、曹操高陵保护和大运河安阳段申遗工作，加快建设中国文字博物馆二期和中国考古博物馆。规划建设殷商·洹河文化旅游、博物馆·会展文化等10个文化产业集聚区和华夏历史文明传承创新区标志性主题公园。加强文化资源整合，加快培育大型文化企业集团。鼓励支持文化创意、数字电视、动漫等新兴文化产业发展，大力发展民俗文化，推出以反映殷商文化、建安文化等为主题的电视剧和演艺剧。完善公共文化服务体系，积极实施文化惠民工程。积极发展旅游业。围绕打造国际旅游城，大力实施“十系百项”行动计划。组织好中国·安阳殷商文化旅游节、中国（安阳）国际航空运动旅游节、内黄红枣文化旅游节等活动。进一步加强景区管理和基础设施建设，着力打造殷墟、羑里城、红旗渠、太行大峡谷等旅游知名品牌。积极推进航空运动之都建设，加快发展低空经济，筹划建设通用航空产业园区。大力发展现代服务业。规划建设商务中心区，支持各城区和有条件的县（市）规划建设特色商务区（街），形成一批服务业产业集群。积极发展金融、房地产等现代服务业，重点发展生产性物流和第三方物流。加强信息网络设施建设，推动“无线城市”应用，提升信息化水平。争取年内催生3家当地新型金融机构，引进3家非银行金融机构，支持符合条件的企业上市融资。

    （七）以改革创新为动力，不断增强经济发展活力。围绕破除制约科学发展体制机制障碍，坚定不移地深化重点领域和关键环节改革，构建有利于科学发展的体制机制。深入推进各项改革。继续深化企业改革，用两年时间完成国企改革扫尾工作，完成市直机关所办企业脱钩改制工作。加强企业国有资产监管。加快推进产业整合，打造一批行业领军企业。深化文化企事业单位改革，巩固提高文化单位转企改制成果。完善文化市场综合执法改革。加快民营经济和中小企业发展。深入推进集体林权制度改革。继续抓好农村综合改革。积极推进事业单位分类改革。深入推进水冶镇行政管理体制改革试点。创新投融资体制机制。完善政府投融资平台体系，推动政府资源向优势平台公司集中，提高信用级别，增强融资能力。深入推进与各商业银行的战略合作，积极推动银企对接，引导更多的社会资金投入实体经济，努力缓解中小企业融资难问题。支持企业上市融资、发行债券和信托融资，积极发展融资租赁、股权投资和风险投资。规范民间融资，加大非法集资打击力度，优化金融生态环境，防范化解金融风险。创新土地开发利用管理机制。按照“三保一高”的要求，完善土地开发利用管理“三项机制”。实施差别化土地政策，重点保障市中心城区重点项目和重要基础设施用地。扎实开展农村土地整治，保障新型农村社区及非农产业发展用地。推动土地承包经营权流转，发展土地规模经营。创新人力资源开发机制。围绕促进就业增收、满足招商引资和产业集聚区用工需求，加快实施全民技能振兴工程，积极开展订单式培训和定向培养。以高层次人才、高技能人才为重点，统筹推进各类人才队伍建设。

    （八）以城市建设管理为关键，全面提升城市综合承载能力。积极开展城乡建设提升行动，以基础设施建设为重点，不断构筑功能完善、协调配套、高效便捷、支撑有力的城市基础设施网络。积极推进基础设施建设。加快城镇交通、供排水、垃圾污水处理、供热、供电、供气、生态等基础设施建设。加强城市整体功能配套衔接，合理布局教育、卫生、文化等公共服务设施和环保、商业服务设施。完善安东新城、安汤新城和新西区配套基础设施。完成文昌大道、永明路、朝阳路等新建路段热力管线建设。积极推进洹北污水处理厂和北小庄污水处理厂建设。加快人民医院搬迁、南水北调调蓄池等项目进度。进一步做好洹河、洪河、茶店河治理，启动羑河治理工程。稳步推进旧城、“城中村”改造。加快推进八中片区、人民大道中段和京港澳高速南北出入口、南大街等区域改造。完成文明大道西段、文源街等道路建设工程。着力提升城市功能品位。全面实施新区公共建筑物、文峰大道西段金融区等标志性建筑和重点区域的“净化、美化、亮化”工程。巩固创建国家卫生城市成果，扎实推进城乡环境卫生整治。积极推进城市水系建设。坚持公交优先，加强公交基础设施建设。积极治理交通拥堵，努力改善居民出行条件。扎实做好数字化城市管理二期工程，努力实现建成区全覆盖。继续抓好“优胜杯”竞赛。全力构建现代综合交通枢纽。京港澳高速文峰立交桥、林长高速主线年内竣工通车，石武高铁配套工程全面完成。开工建设107国道安邯、汤鹤快速通道，加快省道219、228、301升级改造。积极推进鹤辉高速、晋豫鲁铁路通道建设。开工建设新郑机场安阳空港新城一期工程。做好西北环城高速、石林高速、豫东北机场前期工作。

    （九）以民生改善为根本，着力促进社会和谐稳定。加大资金投入，继续实施“十项民生工程”，扎扎实实为人民群众办好一批实事。千方百计扩大就业。加强就业公共服务，重点做好高校毕业生、城镇就业困难人员、退役人员和残疾人就业帮扶工作。实现再就业3万人，其中，就业困难群体再就业1万人。新增农村劳动力转移就业6万人。稳步增加居民收入。继续增加企业退休人员基本养老金，提高城乡低保补助水平，提高优抚对象和困难群众保障标准。增加对农村寄宿制学校和高校食堂的临时性价格补贴。抓好市长负责制的“菜篮子”工程。完善机关事业单位工资制度。加强社会保障体系建设。今年年底前，基本实现新农保和城镇居民社会养老保险制度全覆盖。规范完善失业、医疗、工伤、生育保险市级统筹。大力发展社会福利和慈善事业、妇女儿童事业、老龄事业、残疾人事业。积极推进保障性安居工程建设。新开工廉租住房4600套，经济适用房和限价商品房5100套，公共租赁住房9000套，城市棚户区改造4000户，工矿棚户区改造81户，垦区危旧房改造70户。全面推进各项社会事业。继续实施学前教育三年行动计划，强力推进职业教育攻坚工作，确保安阳师范学院人文管理学院顺利通过教育部本科院校评审。进一步提高公共卫生服务和基本医疗保障水平，促进新增卫生资源重点向基层和农村倾斜。大力开展全民健身活动，积极实施老年体育“长青工程”，办好市第二届全民健身大会。持续稳定低生育水平，促进人口长期均衡发展。加强和创新社会管理。大力推进政务诚信、商务诚信、社会诚信和司法公信建设。强化安全生产监管，有效防范和坚决遏制各类安全事故发生。加强食品药品安全。深入推进普法依法治理工作。深化平安安阳建设。畅通拓宽信访渠道，切实解决人民群众实际问题。坚持科技强警，着力提高打防管控能力，确保社会大局稳定。

    大力弘扬红旗渠精神和新时期“三平”精神，积极争创全国文明城市。加强国防教育，支持驻安解放军、武警部队、人民防空和民兵预备役建设，促进军民融合发展。深入开展“双拥”共建，巩固发展军政军民团结的良好局面。广泛听取各民主党派的意见，发挥工会、共青团、妇联等人民团体的桥梁纽带作用。进一步加强老区建设。继续做好统计、审计、档案、史志、气象、地震、发展研究、外事侨务、民族宗教、红十字等工作。

    （十）以转变职能为重点，努力建设人民满意的政府。认真落实“三具两基一抓手”，深入推进“两转两提”，加强学习型政府、服务型政府、法治型政府、廉洁型政府建设，持续提升政府公信力和执行力。深入推进依法行政。严格执行人大及其常委会的决议决定，依法接受其法律监督和工作监督，主动接受人民政协的民主监督，认真办理人大代表议案、建议和政协委员提案。健全行政监督体系，加强审计监督、行政监察和行政层级监督，规范行政执法行为。加强行政复议，强化行政执法责任和过错追究。建立健全政府决策机制，进一步提高依法决策、科学决策和民主决策水平。不断提高行政效率。推进政企、政事、政府与市场中介组织分开，履行经济调节和市场监管职能，为各类市场主体提供优质服务。强化社会管理和公共服务，维护社会公平正义。加大政务公开，积极推进财政预决算公开。持续开展创先争优活动，加强机关作风建设，努力建设一支政治坚定、业务精湛、作风过硬、人民满意的公务员队伍。深化行政审批制度改革，推动政府工作有序高效运转。着力推动务实发展。深入开展“走基层、听民声”活动，注重调查研究，力戒官僚主义、形式主义。完善重大事项和重点工作跟踪落实机制，确保政令畅通、令行禁止。精简会议和文件，减少迎来送往，严格控制和规范各种检查、评比、达标活动。以务实重干求发展，以务实重干谋跨越，以务实重干求突破。切实做到从严治政。认真落实党风廉政建设目标责任制，严格执行廉洁从政各项规定，着力完善教育、制度、监督、问责并重的惩治和预防腐败体系，强化对权力运行的制约和监督。加大纠风和专项治理力度，切实解决损害群众利益的突出问题，确保人民赋予的权力真正为人民谋利益。严格控制行政经费等一般性支出，坚决反对讲排场、比阔气、铺张浪费等奢靡之风。以廉洁勤政、务实高效的政府形象服务于民、取信于民、造福于民！

    各位代表！

    困难激励斗志，耕耘收获果实。人民的信任和重托，是我们克难攻坚的强大动力。让我们紧密团结在以胡锦涛同志为总书记的党中央周围，在省委、省政府和市委的坚强领导下，解放思想，抢抓机遇，同心同德，励精图治，奋力谱写安阳跨越发展的新篇章，以优异成绩迎接党的十八大胜利召开！
